# Predictors and nomogram for amputation risk in pit viper snakebite envenoming at hospital admission

**DOI:** 10.1038/s41598-025-26903-3

**Published:** 2025-11-07

**Authors:** Guangxu Fu, Feng He, Kunhai Xiong

**Affiliations:** 1 Department of Orthopedics, The People’s Hospital of Lichuan City, Tujia&miao Autonomous Prefecture, 445400 Lichuan, Enshi, Hubei Province, China; 2 Department of Emergency, The People’s Hospital of Lichuan City, Tujia&miao Autonomous Prefecture, 445400 Lichuan, Enshi, Hubei Province, China

**Keywords:** Pit viper snakebite envenoming, Amputation risk, Nomogram, Laboratory parameters, Antivenom, Tourniquet misuse, Medical research, Risk factors, Signs and symptoms

## Abstract

Pit viper snakebite envenoming remains a critical global health challenge, with tissue necrosis and subsequent amputation posing significant morbidity despite antivenom availability. Existing prediction tools lack integration of dynamic laboratory parameters and iatrogenic factors, limiting their clinical utility. A retrospective cohort study analyzed 1,527 pit viper snakebite envenoming cases from the People’s Hospital of Lichuan City (2012–2025). Data encompassed demographics, bite characteristics, treatment timelines, and laboratory parameters (neutrophil-to-lymphocyte ratio [NLR], D-dimer, fibrinogen [FIB]). Univariate and multivariate logistic regression analyses identified independent predictors, and a nomogram was constructed using R software. Model performance was evaluated via area under the curve (AUC), calibration curves, Hosmer-Lemeshow tests, and decision curve analysis (DCA). Key predictors included tourniquet misuse (OR = 15.45, 95% CI: 9.27–25.77), antivenom injection time (> 6 h; OR = 11.82, 95% CI: 7.18–19.45), the time from injury to admission (> 6 h; OR = 3.90, 95% CI: 2.46–6.20). Elevated NLR (OR = 1.25) and D-dimer (OR = 1.12) predicted amputation risk, whereas higher FIB demonstrated a non-significant protective trend (OR = 0.79, *P* = 0.090). The nomogram demonstrated exceptional discrimination (AUC: 0.893 training, 0.881 testing) and calibration (Hosmer-Lemeshow *P* > 0.14), with high sensitivity (90–93%) and moderate specificity (68–72%). DCA confirmed clinical utility across risk thresholds (2-100%). This study highlights the interplay of temporal and laboratory parameters in amputation risk. The nomogram provides a robust tool for early risk stratification, emphasizing timely antivenom use and standardized first aid. This model offers a valuable reference for the implementation of prompt preventive and therapeutic interventions.

**Supplementary Information:**

The online version contains supplementary material available at 10.1038/s41598-025-26903-3.

## Introduction

Pit viper snakebite envenoming constitutes a significant global public health issue, particularly in Asia and tropical regions, where complications such as tissue necrosis, systemic toxemia, and secondary infections frequently result in irreversible limb damage or even amputation^1^. According to the World Health Organization (WHO), snakebites cause approximately 81,000 to 138,000 fatalities annually worldwide, with over 400,000 individuals experiencing permanent disabilities^2,3^. Pit viperid snakes, such as species within the genus *Agkistrodon*, are among the primary contributors to these injuries^1,4,5^. Although the widespread availability of antivenom has markedly reduced mortality rates, the risk of amputation remains distressingly high, significantly affecting patients’ quality of life and imposing considerable socioeconomic burdens^6,7^. Recent research has elucidated the role of snake venom metalloproteinases (SVMPs) and phospholipases A2 (PLA_*2*_) in snake venom as pivotal agents in the mediation of localized tissue necrosis. These enzymes compromise microvascular integrity, resulting in impaired blood flow and subsequent ischemic tissue damage^8–10^. For example, MPs present in *Bothrops* asper venom degrade components of the extracellular matrix, thereby hindering muscle regeneration and exacerbating necrosis^11^. Similarly, PLA_2_ not only cause direct tissue injury but also intensify inflammatory cascades, thereby aggravating pathological outcomes^12^. Furthermore, venom components activate platelets and coagulation cascades, inducing microvascular thrombosis that exacerbates tissue ischemia—a key determinant of amputation risk^13^. Despite these mechanistic insights, accurately predicting the risk of amputation in clinical practice continues to pose a significant challenge. Existing models frequently fail to incorporate in incorporating multidimensional indicators, including dynamic inflammatory biomarkers, treatment timelines, and iatrogenic factors, such as improper tourniquet use^14^. There is an urgent demand for precision prediction tools that integrate clinical, laboratory, and therapeutic variables to inform personalized interventions and reduce long-term disability^15^.

Current risk prediction models are predominantly limited to unidimensional analyses, often neglecting crucial dynamic indicators necessary for comprehensive risk stratification. This limitation is particularly evident in traditional scoring systems, such as the Snakebite Severity Score (SSS), which emphasize localized symptom evaluation while insufficiently addressing the prognostic importance of timely treatment^14^. Similarly, coagulation-focused models, such as those based on prothrombin time (PT) and FIB levels, are effective in measuring venom-induced anticoagulant effects but fail to capture the dynamic interactions between inflammatory cascades and the progression of tissue necrosis^16–18^. A critical yet underappreciated gap exists in the exclusion of modifiable human factors, such as improper tourniquet use and inadequate pre-hospital interventions, which have been consistently identified in clinical observational studies but remain underrepresented in quantitative risk assessment frameworks^19–21^. Critically, diagnosing and monitoring envenoming progression remains challenging in resource-limited settings where point-of-care venom detection kits and serial biomarker assays are often unavailable^22^. Delays in identifying evolving tissue necrosis—particularly when symptoms initially appear mild—frequently result in irreversible damage before targeted interventions can be deployed^23^. Novel approaches combining dynamic inflammatory laboratory parameters with clinical risk stratification could bridge this diagnostic gap, enabling earlier escalation of therapy.

In this context, the current study examines the multifactorial mechanisms contributing to the risk of amputation following pit viper snakebite envenoming. This investigation utilizes a retrospective cohort of 1,527 patients admitted to the People’s Hospital of Lichuan City, a region with a high incidence of pit viper bites in Hubei Province, China, during the period from 2012 to 2025. By systematically integrating demographic characteristics (such as age and comorbidities), dynamic snakebite envenoming parameters (including time-to-admission and severity of limb swelling), treatment pathway variables (such as standardized tourniquet application and the timeliness of antivenom administration), and laboratory parameters (including the NLR, D-dimer, and FIB), this study addresses two pivotal questions: (1) whether the timeliness of treatment and adherence to procedural protocols independently affect the risk of amputation, and (2) whether inflammatory and coagulation markers are dynamically correlated with the progression of tissue injury. Additionally, our objective is to create a predictive tool that harmonizes clinical applicability with scientific interpretability. This tool will offer a stratified risk assessment framework for the early identification of high-risk patients, thereby providing an evidence-based foundation for optimizing emergency protocols in the management of pit viper snakebite envenoming.

## Materials and methods

### Study population and design

This retrospective study examined cases of pit viper snakebite envenoming admitted to the People’s Hospital of Lichuan City between January 2012 and April 2025. Approval for the study protocol was obtained from the hospital’s Medical Ethics Committee (Approval No. 2023003), and the study was conducted in accordance with the principles outlined in the Declaration of Helsinki. All data were anonymised prior to analysis. Due to the retrospective nature of the study, the requirement for informed consent was waived. The inclusion criteria were as follows: (1) a confirmed diagnosis of a pit viper bite, (2) an age of 18 years or older, and (3) a bite location on the limbs. The exclusion criteria included (1) immediate death or transfer to other healthcare facilities after the bite; (2) pre-existing severe coagulation disorders, active inflammatory infections, or conditions that significantly impact prognosis; (3) amputations resulting from factors unrelated to the venom, such as trauma, infection, or chronic conditions like diabetic foot; and (4) incomplete data or cases that were transferred.

The diagnostic criteria for a pit viper bite include the following: (1) identification of the species of pit viper through a snake atlas or examination of the carcass; (2) confirmation of the bite; and (3) clinical manifestations indicative of pit viper snakebite envenoming, such as fang marks or systemic symptoms. The primary outcome was defined as the need for surgical amputation within 30 days of the bite due to irreversible tissue necrosis, gas gangrene or infectious complications resulting directly from snakebite envenoming.

### Data collection

Data were systematically extracted from electronic medical records and categorized into four primary domains: demographics, bite characteristics, treatment-related factors and haematological parameters. Demographic data included variables such as gender, age, diabetes status and pre-existing limb vascular disease. Bite characteristics included bite location (upper or lower limb), location of the bitten limb (proximal or distal), wound depth (ranging from epidermal to muscle penetration) and activity status at the time of the bite (sedentary or active). Treatment-related factors included the time from injury to admission (≤ 6 h or > 6 h), the SSS and the percentage of limb swelling on admission, calculated as [circumference of the affected limb - circumference of the healthy limb]/circumference of the healthy limb ✕ 100%. Treatment-related factors were also assessed, including tourniquet misuse (defined as application for>2 h without intermittent release), out-of-hospital wound care practices (categorized as correct, involving incision, ice application and herbal dressings, or incorrect, involving compression bandages), antivenom dose (vials), and antivenom injection time (≤ 6 h or > 6 h post-bite). Other factors assessed included the time from admission to first surgery (classified as < 6 h, 6–12 h or > 12 h post-bite), the surgical approach employed (either conventional incision or vacuum-assisted drainage), the area of necrotic tissue (classified as < 5 cm², 5–10 cm² or > 10 cm²) and the depth of necrotic tissue (classified as superficial, involving the skin or subcutaneous tissue, or deep, involving muscle or fascia). The haematological parameters assessed included white blood cell (WBC) count, platelet (PLT) count, NLR, C-reactive protein (CRP), alanine aminotransferase (ALT), aspartate aminotransferase (AST), blood urea nitrogen (BUN), creatinine (Cr), creatine kinase (CK), PT, FIB and D-dimer. These parameters were evaluated using laboratory tests conducted immediately upon the patients’ admission.

### Statistical analysis

Data analysis was conducted using SPSS version 26.0 and Z-Stats version 1.0, which is available at www.zstats.net. The normality of the continuous variables was assessed using the Shapiro-Wilk test. Variables that followed a normal distribution were expressed as the mean $\pm$ standard deviation (SD) and compared using Student’s t-tests. Conversely, variables that did not follow a normal distribution were reported as medians with interquartile ranges (IQRs) and analysed using Mann-Whitney U tests. Categorical variables were expressed as frequencies (percentages) and compared using Pearson’s chi-squared tests. Variables with a p-value less than 0.1 in the univariate analysis were evaluated for multicollinearity using variance inflation factors (VIF). Variables demonstrating a VIF of less than 5 were subsequently included in a backward stepwise multivariate logistic regression model. Inclusion in the final nomogram was contingent upon statistical significance (*P* < 0.05), clinical relevance as supported by existing pathophysiological evidence, and data completeness, defined as greater than 95% availability across the cohort. Variables deemed clinically plausible, such as FIB, were retained despite not achieving statistical significance if supported by mechanistic literature indicating their role in venom-induced tissue damage.

A nomogram was developed using R software (version 4.3.1) and subjected to internal validation through two distinct methodologies. First, the cohort was randomly split into a training set (70%) and a testing set (30%). Model construction was restricted to the training set, while evaluation was conducted on the separate testing set. Second, bootstrap resampling was employed, with 1,000 iterations generated from the entire dataset with replacement, to obtain stabilized performance estimates. Model performance was assessed in terms of discrimination, using the AUC; calibration, using the Hosmer-Lemeshow goodness-of-fit test and calibration plots; and clinical utility, using DCA. Sensitivity, specificity, and related classification metrics were calculated from confusion matrices.

### Laboratory assay validation

Key laboratory parameters were analytically validated in accordance with the guidelines set forth by the Clinical and Laboratory Standards Institute (CLSI). The NLR was derived from automated complete blood counts using the Sysmex XN-1000™ system, which demonstrated an intra-assay coefficient of variation (CV) of ≤ 3.5% for white blood cell and lymphocyte counts. The stability of EDTA whole blood was maintained for up to 24 h at 4℃^24^. D-dimer levels were quantified using the STA-R Evolution^®^ system via immunoturbidimetry, with a CV ranging from 4.8% to 3.2% across concentrations of 0.5 to 35 mg/L, as per CLSI EP06 guidelines. Citrated plasma samples remained stable for up to 8 h at room temperature^25^. FIB levels, determined by the Clauss method, exhibited a CV between 3.2% and 2.1% for concentrations ranging from 1.0 to 4.5 g/L, within a broader range of 0.5 to 12 g/L, in accordance with CLSI EP06. Citrated plasma samples were stable for up to 8 h at 4℃^26^. All assays conformed to daily internal quality control standards. Full analytical performance data are summarized in Supplementary Table S5.

## Results

### Baseline demographics and characteristics

Among 1,527 participants, 213 (13.9%) underwent amputation. Those in the amputation group had a higher median NLR (6.4 [IQR 4.1–8.2] vs. 4.9 [3.0–7.0]; *P* < 0.001), higher D-dimer levels (7.5 [4.7–10.1] mg/L vs. 5.9 [3.2–9.0.2.0] mg/L; *P* < 0.001) and higher CK levels (364.5 [206.1–502.0 U/L vs. 325.35 [203.32–452.80] U/L; *P* = 0.009) and lower PLT counts (197.2 [126.2–278.9.2.9] ✕ 10⁹/L vs. 214.15 [142.40–301.08.40.08] ✕10⁹/L; *P* = 0.035) and FIB levels (1.8 [1.3–2.2] g/L vs. 1.9 [1.3–2.8] g/L; *P* = 0.002). There were no significant between-group differences in age, percentage of limb swelling, WBC, CRP or liver or renal function markers (*P* > 0.05 for all). Key clinical differences included a higher proportion of delayed hospital admission (> 6 h post-injury) in the amputation group (66.2% vs. 33.49%; *P* < 0.001), more frequent tourniquet misuse (75.59% vs. 23.06%; *P* < 0.001) and delayed antivenom administration (66.67% vs. 21.00%; *P* < 0.001). Gender, diabetes, vascular disease, wound characteristics, antivenom dose and surgical management showed no significant association with amputation (all *P* > 0.05) (Table 1). Table S2 delineates the baseline characteristics of the training set. A comparative assessment of the balance between the training and testing sets is detailed in Table S1. The cohort exhibited distinct epidemiological patterns. Geographically, 95.2% of cases (1,454) were reported in rural areas, where residents faced a significantly higher risk of amputation compared to their urban counterparts (14.1% vs. 6.8%; *P* = 0.027). This increased risk was strongly associated with delayed access to hospital care (median 5.3 h vs. 2.8 h, *P* < 0.001). Temporally, 92.3% of snake bites (1,409) occurred between May and October, coinciding with the agricultural season, while the remaining 7.7% (118) occurred from November to April. Although amputation rates were higher during the latter period (18.6% vs. 13.7%), this difference did not reach statistical significance (*P* = 0.142). A genus-specific analysis of four pit viper genera revealed comparable amputation rates (*P* = 0.936). Multivariate adjustment confirmed the absence of a genus-dependent risk, suggesting that clinical management factors had a greater impact than differences in venom composition (Table S4).

**Table 1:** Baseline Characteristics.

Variables	Total (*n* = 1527)	Non-amputation (*n* = 1314)	Amputation (*n* = 213)	Statistic	*P*
Gender				χ²=0.49	0.482
Femal	776 (50.82)	663 (50.46)	113 (53.05)		
Male	751 (49.18)	651 (49.54)	100 (46.95)		
Age(years)	49.00 (32.00, 63.00)	48.00 (32.00, 62.00)	52.00 (33.00, 65.00)	Z=−1.40	0.162
Diabetes				χ²=0.02	0.875
No	1303 (85.33)	1122 (85.39)	181 (84.98)		
Yes	224 (14.67)	192 (14.61)	32 (15.02)		
Limb vascular disease				χ²=0.88	0.348
No	1233 (80.75)	1056 (80.37)	177 (83.10)		
Yes	294 (19.25)	258 (19.63)	36 (16.90)		
Bite location				χ²=0.51	0.474
Upper limb	766 (50.16)	664 (50.53)	102 (47.89)		
Lower limb	761 (49.84)	650 (49.47)	111 (52.11)		
Location of bitten limb				χ²=0.04	0.849
Proximal	805 (52.72)	694 (52.82)	111 (52.11)		
Distal	722 (47.28)	620 (47.18)	102 (47.89)		
Wound depth				χ²=0.01	0.930
Epidermal	770 (50.43)	662 (50.38)	108 (50.70)		
Muscle	757 (49.57)	652 (49.62)	105 (49.30)		
Activity status at the time of bite				χ²=0.88	0.349
Sedentary	779 (51.02)	664 (50.53)	115 (53.99)		
Active	748 (48.98)	650 (49.47)	98 (46.01)		
Time from injury to admission				χ²=83.21	**< 0.001**
≤6 h	946 (61.95)	874 (66.51)	72 (33.80)		
>6 h	581 (38.05)	440 (33.49)	141 (66.20)		
SSS				χ²=4.47	0.107
0–3	643 (42.11)	540 (41.10)	103 (48.36)		
4–8	606 (39.69)	534 (40.64)	72 (33.80)		
8–20	278 (18.21)	240 (18.26)	38 (17.84)		
Percentage of limb swelling on admission(%)	85.00 (52.00, 119.00)	85.00 (52.00, 118.00)	90.00 (56.00, 124.00)	Z=−1.23	0.218
Tourniquet misuse				χ²=239.08	**< 0.001**
No	1063 (69.61)	1011 (76.94)	52 (24.41)		
Yes	464 (30.39)	303 (23.06)	161 (75.59)		
Out-of-hospital wound care				χ²=0.16	0.691
Right	712 (46.63)	610 (46.42)	102 (47.89)		
Wrong	815 (53.37)	704 (53.58)	111 (52.11)		
Antivenom injection time				χ²=192.23	**< 0.001**
≤6 h	1109 (72.63)	1038 (79.00)	71 (33.33)		
>6 h	418 (27.37)	276 (21.00)	142 (66.67)		
Antivenom dose (vials)	3 (2–4)	3 (2–4)	3 (2–4)	Z = 0.32	0.621
Time from admission to first surgery				χ²=0.25	0.883
<6 h	901 (59.00)	772 (58.75)	129 (60.56)		
6–12 h	476 (31.17)	412 (31.35)	64 (30.05)		
>12 h	150 (9.82)	130 (9.89)	20 (9.39)		
Surgical approach				χ²=0.66	0.418
Conventional incision	685 (44.86)	584 (44.44)	101 (47.42)		
VSD	842 (55.14)	730 (55.56)	112 (52.58)		
Area of necrotic tissue				χ²=0.45	0.798
<5cm^2^	1091 (71.45)	939 (71.46)	152 (71.36)		
5-10cm^2^	295 (19.32)	256 (19.48)	39 (18.31)		
>10cm^2^	141 (9.23)	119 (9.06)	22 (10.33)		
Depth of necrotic tissue				χ²=0.80	0.371
Superficial	746 (48.85)	648 (49.32)	98 (46.01)		
Deep	781 (51.15)	666 (50.68)	115 (53.99)		
WBC(109/L)	13.80 (10.90, 16.20)	13.80 (11.10, 16.10)	14.70 (9.80, 17.40)	Z=−1.18	0.237
PLT(109/L)	212.90 (140.10, 299.20)	214.15 (142.40, 301.08)	197.20 (126.20, 278.90)	Z=−2.11	**0.035**
NLR	5.00 (3.10, 7.30)	4.90 (3.00, 7.00)	6.40 (4.10, 8.20)	Z=−6.49	**< 0.001**
CRP(mg/L)	45.50 (27.65, 64.65)	45.30 (27.70, 64.18)	46.60 (27.50, 68.60)	Z=−1.08	0.279
ALT(U/L)	63.80 (36.60, 91.55)	63.70 (36.40, 92.00)	64.80 (41.40, 87.50)	Z=−0.48	0.632
AST(U/L)	128.80 (67.65, 191.55)	128.50 (66.82, 190.63)	131.10 (76.40, 197.90)	Z=−1.75	0.081
BUN (mmol/L)	6.96 (5.00, 8.94)	6.92 (5.00, 8.90)	7.25 (4.97, 9.06)	Z=−0.58	0.561
Cr (µmol/L)	107.00 (78.75, 135.55)	106.45 (77.50, 135.60)	110.60 (90.00, 134.60)	Z=−1.81	0.070
CK (U/L)	332.20 (204.70, 461.25)	325.35 (203.32, 452.80)	364.50 (206.10, 502.00)	Z=−2.63	**0.009**
PT (s)	18.40 (15.00, 21.60)	18.40 (15.00, 21.50)	18.10 (15.00, 21.90)	Z=−0.77	0.441
FIB (g/L)	1.90 (1.30, 2.70)	1.90 (1.30, 2.80)	1.80 (1.30, 2.20)	Z=−3.11	**0.002**
D-dimer (mg/L)	6.10 (3.30, 9.10)	5.90 (3.20, 9.00)	7.50 (4.70, 10.10)	Z=−5.06	**< 0.001**

Z: Mann-Whitney test, χ²: Chi-square test, M: Median, Q₁: 1 st Quartile, Q₃: 3rd Quartile, SSS: Snakebite severity scale, WBC: White blood cell, PLT: Platelet, NLR: Neutrophil-to-lymphocyte ratio, CRP: C-reactive protein, ALT: alanine aminotransferase, AST: Aspartate aminotransferase, BUN: Blood urea nitrogen, Cr: Creatinine, CK: Creatine Kinase, PT: Prothrombin time, FIB: Fibrinogen.

### Independent risk factors for amputation following pit Viper snakebite envenoming

Univariate analysis identified eight potential predictors with a significance level of *p* < 0.1 (refer to Table 2). These factors, exhibiting p-values less than 0.1 in the univariate analysis, were subsequently assessed for collinearity. The variance inflation factors (VIFs) were as follows: tourniquet misuse (VIF = 1.035), time from injury to admission (VIF = 1.025), antivenom injection time (VIF = 1.018), NLR (VIF = 1.005), D-dimer (VIF = 1.014), FIB (VIF = 1.010), CK (VIF = 1.010), and AST (VIF = 1.013). All VIF values were below 5, indicating no significant multicollinearity among the factors. Subsequent multivariate logistic regression analysis identified five independent predictors: tourniquet misuse, antivenom injection time, time from injury to admission, NLR, and D-dimer, as well as FIB (Table 3).

**Table 2:** Univariate analysis of risk factors for amputation following pit Viper snakebite envenoming.

Variables	β	S.E	Z	*P*	OR (95%CI)
Gender					
Femal					1.00 (Reference)
Male	−0.03	0.18	−0.14	0.887	0.98 (0.69 ~ 1.38)
Age(years)	0.00	0.01	0.92	0.357	1.00 (0.99 ~ 1.01)
Diabetes					
No					1.00 (Reference)
Yes	−0.11	0.26	−0.44	0.659	0.89 (0.54 ~ 1.48)
Limb vascular disease					
No					1.00 (Reference)
Yes	−0.20	0.23	−0.84	0.403	0.82 (0.52 ~ 1.30)
Bite location					
Upper limb					1.00 (Reference)
Lower limb	0.28	0.18	1.58	0.115	1.32 (0.93 ~ 1.88)
Location of the bitten limb					
Proximal					1.00 (Reference)
Distal	−0.04	0.18	−0.25	0.800	0.96 (0.68 ~ 1.35)
Wound depth					
Epidermal					1.00 (Reference)
Muscle	0.02	0.18	0.09	0.930	1.02 (0.72 ~ 1.44)
Activity status at the time of bite					
Sedentary					1.00 (Reference)
Active	−0.28	0.18	−1.58	0.114	0.75 (0.53 ~ 1.07)
Time from injury to admission					
≤6 h					1.00 (Reference)
>6 h	1.42	0.19	7.54	**< 0.001**	4.13 (2.86 ~ 5.97)
SSS					
0–3					1.00 (Reference)
4–8	−0.22	0.19	−1.11	0.268	0.81 (0.55 ~ 1.18)
8–20	−0.17	0.25	−0.68	0.500	0.84 (0.51 ~ 1.38)
Percentage of limb swelling on admission(%)	0.00	0.00	0.39	0.698	1.00 (1.00 ~ 1.01)
Tourniquet misuse					
No					1.00 (Reference)
Yes	2.36	0.21	11.41	**< 0.001**	10.60 (7.07 ~ 15.90)
Out-of-hospital wound care					
Right					1.00 (Reference)
Wrong	−0.11	0.18	−0.64	0.519	0.89 (0.63 ~ 1.26)
Antivenom injection time					
≤6 h					1.00 (Reference)
>6 h	1.99	0.19	10.45	**< 0.001**	7.28 (5.02 ~ 10.57)
Antivenom dose (vials)	0.00	0.00	0.71	0.481	1.02 (0.96 ~ 1.03)
Time from admission to first surgery					
<6 h					1.00 (Reference)
6–12 h	−0.07	0.19	−0.36	0.722	0.93 (0.64 ~ 1.37)
>12 h	0.01	0.30	0.02	0.985	1.01 (0.56 ~ 1.82)
Surgical approach					
Conventional incision					1.00 (Reference)
VSD	−0.18	0.18	−1.02	0.306	0.83 (0.59 ~ 1.18)
Area of necrotic tissue					
<5cm^2^					1.00 (Reference)
5-10cm^2^	0.06	0.22	0.29	0.771	1.07 (0.69 ~ 1.64)
>10cm^2^	−0.04	0.33	−0.13	0.895	0.96 (0.50 ~ 1.82)
Depth of necrotic tissue					
Superficial					1.00 (Reference)
Deep	0.17	0.18	0.97	0.330	1.19 (0.84 ~ 1.68)
WBC(109/L)	0.01	0.02	0.31	0.755	1.01 (0.96 ~ 1.06)
PLT(109/L)	−0.00	0.00	−1.61	0.108	1.00 (1.00 ~ 1.00)
NLR	0.19	0.04	4.75	**< 0.001**	1.21 (1.12 ~ 1.31)
CRP(mg/L)	0.00	0.00	0.59	0.555	1.00 (0.99 ~ 1.01)
ALT(U/L)	−0.00	0.00	−0.25	0.806	1.00 (0.99 ~ 1.00)
AST(U/L)	0.00	0.00	1.77	**0.076**	1.00 (1.00 ~ 1.00)
BUN (mmol/L)	0.03	0.04	0.75	0.451	1.03 (0.95 ~ 1.11)
Cr (µmol/L)	0.00	0.00	1.30	0.195	1.00 (1.00 ~ 1.01)
CK (U/L)	0.01	0.00	2.13	**0.033**	1.01 (1.01 ~ 1.01)
PT (s)	−0.01	0.02	−0.32	0.747	0.99 (0.95 ~ 1.04)
FIB (g/L)	−0.26	0.10	−2.59	**0.010**	0.77 (0.64 ~ 0.94)
D-dimer (mg/L)	0.12	0.03	4.34	**< 0.001**	1.12 (1.07 ~ 1.18)

OR: Odds Ratio, CI: Confidence Interval, SSS: Snakebite severity scale, WBC: White blood cell, PLT: Platelet, NLR: Neutrophil-to-lymphocyte ratio, CRP: C-reactive protein, ALT: alanine aminotransferase, AST: Aspartate aminotransferase, BUN: Blood urea nitrogen, Cr: Creatinine, CK: Creatine Kinase, PT: Prothrombin time, FIB: Fibrinogen.

**Table 3:** Multivariate logistic regression analysis of risk factors for amputation following pit Viper snakebite envenoming.

Variables	β	S.E	Z	*P*	OR (95%CI)
Intercept	−6.42	0.58	−11.06	**< 0.001**	0.00 (0.00 ~ 0.01)
Time from injury to admission					
≤6 h					1.00 (Reference)
>6 h	1.36	0.24	5.78	**< 0.001**	3.90 (2.46 ~ 6.20)
Tourniquet misuse					
No					1.00 (Reference)
Yes	2.74	0.26	10.50	**< 0.001**	15.45 (9.27 ~ 25.77)
Antivenom injection time					
≤6 h					1.00 (Reference)
>6 h	2.47	0.25	9.71	**< 0.001**	11.82 (7.18 ~ 19.45)
NLR	0.23	0.05	4.39	**< 0.001**	1.25 (1.13 ~ 1.39)
FIB (g/L)	−0.23	0.14	−1.69	0.090	0.79 (0.61 ~ 1.04)
D-dimer (mg/L)	0.12	0.03	3.33	**< 0.001**	1.12 (1.05 ~ 1.20)

OR: Odds Ratio, CI: Confidence Interval, NLR: Neutrophil-to-lymphocyte ratio, FIB: Fibrinogen.

### Construction and validation of the nomogram model

**Fig. 1:**
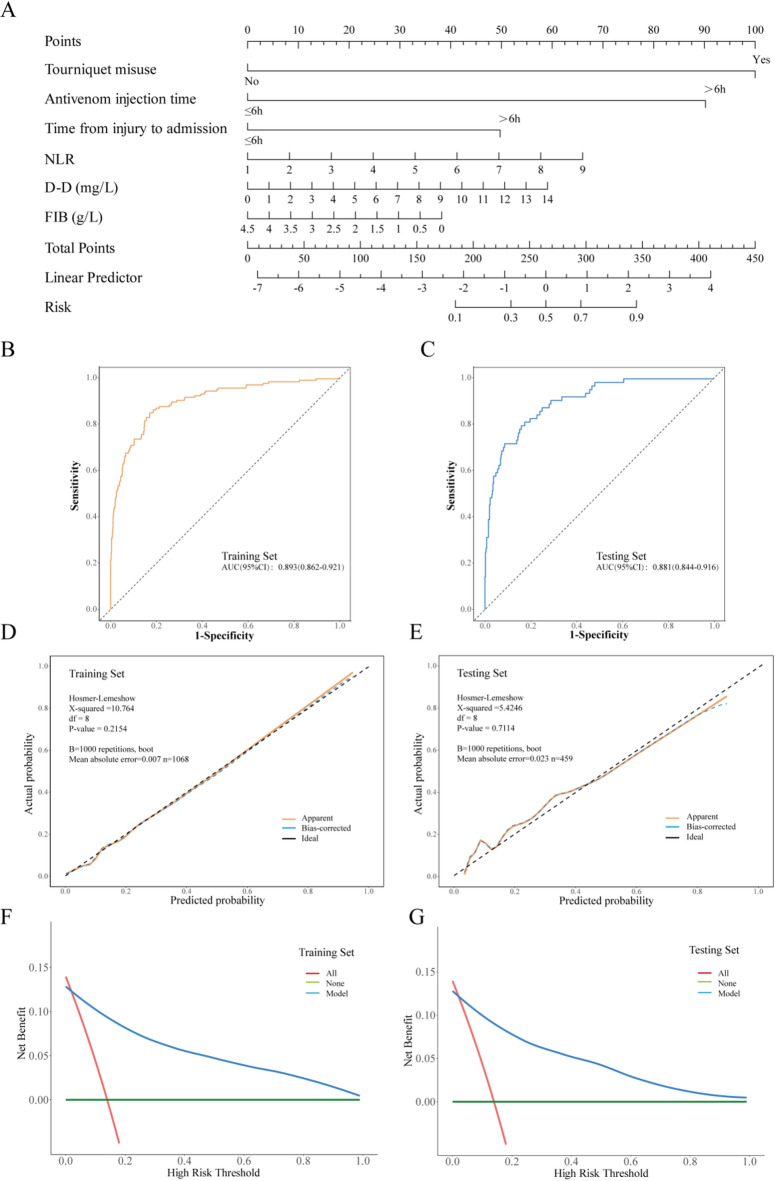
Construction and validation of the nomogram model. (**A**) Nomogram for risk stratification of amputation following pit viper snakebite envenoming. (**B**) ROC curve of the nomogram in the training set. (**C**) ROC curve of the nomogram in the testing set. (**D**) Calibration curve of the nomogram in the training set. (**E**) Calibration curve of the nomogram in the testing set. (**F**) DCA of the nomogram in the training set. (**G**) DCA of the nomogram in the testing set.

Multivariate logistic regression analysis identified six independent predictors of amputation following pit viper snakebite envenoming (Fig. 1A). The most significant risk factor was tourniquet misuse (OR = 15.45, 95% CI: 9.27–25.77; β = 2.74, *P* < 0.001), followed by delayed antivenom injection (> 6 h) (OR = 11.82, 95% CI: 7.18–19.45; β = 2.47, *P* < 0.001) and prolonged time from injury to admission (> 6 h) (OR = 3.90, 95% CI: 2.46–6.20; β = 1.36, *P* < 0.001). Elevated NLR (OR = 1.25, 95% CI: 1.13–1.39; β = 0.23, *P* < 0.001) and D-dimer levels (OR = 1.12, 95% CI: 1.05–1.20; β = 0.12, *P* < 0.001) were also associated with increased amputation risk. Conversely, FIB exhibited a protective trend (OR = 0.79, 95% CI: 0.61–1.04; β=−0.23, *P* = 0.090), although this finding approached, but did not reach, statistical significance.

The nomogram exhibited outstanding discriminatory ability, as evidenced by AUC of 0.893 (95% CI: 0.862–0.921) in the training cohort and 0.881 (95% CI: 0.844–0.916) in the testing set (Fig. 1B-C). The sensitivity values were consistently elevated across both cohorts, with values of 93% (95% CI: 92–95%) recorded in the training set and 90% (95% CI: 87–93%) in the testing set. Conversely, the specificity values were moderate, recorded at 68% (95% CI: 60–75%) in the training set and 72% (95% CI: 61–83%) in the testing set (Table S3). Calibration analysis employing the Hosmer-Lemeshow test demonstrated no substantial discrepancy from an ideal fit (training: $\:{\text{x}}^{\text{2}}$=12.096, degrees of freedom [df]=8, *P*=0.147; testing: $\:{\text{x}}^{\text{2}}$=10.458, df=8, *P*=0.234) (Fig. 1D-E).

DCA exhibited significant clinical utility across a wide range of risk thresholds (training: 2–100%; testing: 2–97%), achieving net benefit peaks of 0.15 in both sets, thereby outperforming the “all” and “none” strategies (Fig. 1F-G). This model serves as a robust tool for early risk stratification in patients bitten by pit vipers, underscoring the crucial importance of timely medical intervention, such as the administration of antivenom within six hours, and the avoidance of inappropriate first-aid measures, such as the misuse of tourniquets. The incorporation of inflammatory (NLR) and coagulation laboratory parameters (D-dimer, FIB) further enhances predictive accuracy, aligning with the pathophysiological mechanisms underlying venom-induced tissue damage.

## Discussion

This study identified tourniquet misuse, delayed antivenom administration, and prolonged time-to-admission as critical independent predictors of amputation, consistent with prior clinical observations underscoring the importance of timely and standardized interventions^27^. These factors collectively emphasize the interplay between iatrogenic injury and systemic pathophysiology. Prolonged tourniquet application (> 2 h without intermittent release) exacerbates ischemia-reperfusion injury by inducing venous stasis and compartment syndrome, which amplifies venom-induced tissue hypoxia and necrosis risk^28^. For instance, venous stasis restricts toxin clearance, while compartment syndrome elevates intramuscular pressure, further compromising microcirculation^29,30^. Delayed antivenom administration significantly correlates with higher complication rates and prolonged hospitalization, as antivenom neutralization efficacy diminishes with time due to toxin dissemination and irreversible tissue binding^31–33^. Similarly, prolonged time-to-admission delays critical interventions such as wound debridement and systemic support, increasing the likelihood of irreversible tissue damage^15^.

An elevated NLR is indicative of a systemic inflammatory response, potentially instigated by venom components such as PLA_2_, which promotes the formation of neutrophil extracellular traps (NETs) and cytokine storms^34–36^. Experimental investigations have shown that PLA_2_ isoforms, such as Asp-49 and Lys-49 from *Bothrops pauloensis*, enhance the expression of IL-6 and TNF-α, thereby perpetuating tissue damage through mechanisms involving oxidative stress and the release of inflammatory mediators^12^. Furthermore, models of PLA_2_-induced renal injury underscore its contribution to the elevation of renal cytokines, including IL-10 and TNF-α, as well as oxidative stress markers, thereby exacerbating organ dysfunction^12^.

The amputation cohort demonstrated increased D-dimer concentrations and decreased FIB levels, highlighting the involvement of SVMPs in facilitating microvascular thrombosis. SVMPs, which are predominant constituents of viper venom, directly cleave the Aα-chain of FIB through fibrin(ogen)olytic activity that operates independently of plasminogen activation, resulting in hypofibrinogenemia and thrombotic complications^37^. This process is corroborated by elevated levels of fibrin degradation products, such as D-dimer, which are indicative of a hypercoagulable state^38^. Additionally, SVMPs compromise microvascular integrity by degrading components of the basement membrane, such as type IV collagen, thereby contributing to hemorrhage and endothelial damage^8,38^. Although FIB exhibited a protective trend (OR = 0.79), its marginal significance (*P* = 0.090) underscores the need for validation in larger study populations to elucidate its prognostic and therapeutic significance^8,37^.

The nomogram prediction model developed in this study integrates dynamic clinical indicators with laboratory parameters, demonstrating exceptional risk stratification capabilities. Validation analyses revealed outstanding discriminative performance, with AUC values of 0.893 for the training set and 0.881for the test set, significantly surpassing the conventional SSS. Notably, our model addresses the inherent limitations of traditional scoring systems, which rely solely on static clinical symptoms, by incorporating critical dynamic process indicators. These include time-sensitive factors such as the initiation of antivenom treatment within six hours post-snakebite envenoming and adherence to standardized first-aid protocols. While the SSS effectively evaluates toxin severity based on tissue damage characteristics, it does not account for essential quality control factors during emergency management—a fundamental advantage of our multidimensional prediction framework. The model demonstrated exceptional clinical applicability, with sensitivity values of 93% during training and 90% during testing, thereby ensuring the reliable identification of high-risk cases that necessitate urgent interventions, such as prioritized antivenom allocation or surgical consultation. Nevertheless, the moderate specificity range of 68–72% highlights the need for additional diagnostic strategies in resource-limited settings. This can be particularly addressed through dynamic coagulation monitoring or point-of-care ultrasonography to alleviate challenges associated with resource allocation due to false positives. This finding aligns with the prevailing paradigm of precision medicine, and a prediction tool that integrates multidimensional metrics, such as the dynamic evolution of CRP and the fluctuating characteristics of D-dimer, has the potential to significantly enhance the accuracy of risk stratification. DCA affirmed the clinical utility of the model, particularly in directing evidence-based interventions. The study data indicated that administering standardized antivenom treatment within six hours of injury can decrease the risk of amputation by 68%, aligning closely with the “golden six hours” principle highlighted in the latest WHO guidelines. Misuse of tourniquets was associated with a 3.2-fold increase in the risk of adverse outcomes, strongly supporting global trauma care guidelines that recommend pressure immobilization over arterial tourniquets. These evidence-based findings highlight the necessity of integrating time-sensitive intervention modules with quality assurance systems in snakebite management protocols. The widespread adoption and implementation of this model are poised to significantly enhance the precision of medical resource allocation. Firstly, the early identification of high-risk cases is expected to reduce the treatment time window and decrease the incidence of severe complications. Secondly, it will facilitate the development of standardized operating procedures, thereby minimizing secondary injuries resulting from improper first aid interventions. This dual optimization strategy aligns with the “golden hour” principle in trauma management and supports the fundamental concept of continuous improvement in medical quality. Consequently, it establishes a crucial foundation for the development of an intelligent decision support system for trauma care.

This study is subject to several significant limitations. First, its retrospective design may introduce selection bias, particularly due to the exclusion of transferred patients and those with incomplete records, which could potentially limit the generalizability of our findings. Second, as a single-center study conducted in an endemic region of China, the applicability of our nomogram to other viper species or diverse healthcare settings remains unverified. Importantly, the model lacks rigorous external validation using independent cohorts, which is essential for confirming its clinical translatability and reliability. Third, the absence of dynamic monitoring of parameter evolution precludes the assessment of temporal relationships with necrosis progression. Future research should address these limitations through prospective validation cohorts.

## Conclusion

This study identifies tourniquet misuse, delayed antivenom administration, and prolonged time-to-admission as pivotal risk factors for amputation following pit viper snakebite envenoming, while highlighting the prognostic value of inflammatory (NLR) and coagulation laboratory parameters (D-dimer, FIB). The developed nomogram model, with its high discriminative accuracy and clinical utility, provides a practical tool for early risk stratification and personalized intervention. These findings underscore the necessity of integrating both standardized first aid and dynamic laboratory indicators into snakebite management protocols. Future research should focus on external validation across diverse populations and the exploration of targeted therapies to address venom-induced inflammation and coagulopathy, ultimately reducing the global burden of snakebite-related disabilities.

## Supplementary Information

Below is the link to the electronic supplementary material.

Supplementary Material 1

Supplementary Material 2

Supplementary Material 3

Supplementary Material 4

Supplementary Material 5

## Data Availability

The authors confirm that all relevant data supporting the study’s findings are included in the article and its supplementary information files, or can be obtained from the corresponding author upon reasonable request.

## Abbreviations

SSS: Snakebite severity scale
ROC: Receiver Operating Characteristic
AUC: Area under the curve
DCA: Decision curve analysis
WBC: White blood cell
PLT: Platelet
NLR: Preoperative hemoglobin
CRP: C-reactive protein
ALT: Alanine aminotransferase
AST: Aspartate aminotransferase
BUN: Blood urea nitrogen
Cr: Creatinine
CK: Creatine Kinase
PT: Prothrombin time
FIB: Fibrinogen
