# Supplementary material for: Predictors and nomogram for amputation risk in pit viper snakebite envenoming at hospital admission
Source: Sci Rep. 2025 Nov 7;15:39082. doi: 10.1038/s41598-025-26903-3 (PMC12594999; doi:10.1038/s41598-025-26903-3)
Supplement: Supplementary file 1 — Supplementary Material 1 [file 41598_2025_26903_MOESM1_ESM.docx]

**Table S3.**

Confusion Matrix for Training and Testing Sets

| Data | AUC (95%CI) | Accuracy (95%CI) | Sensitivity (95%CI) | Specificity (95%CI) | PPV (95%CI) | NPV (95%CI) | cut off |
| --- | --- | --- | --- | --- | --- | --- | --- |
|  |  |  |  |  |  |  |  |
| Train | 0.90 (0.88-0.93) | 0.90 (0.88-0.92) | 0.93 (0.92 - 0.95) | 0.68 (0.60 - 0.75) | 0.95 (0.93 - 0.96) | 0.62 (0.55 - 0.70) | 0.3 |
| Test | 0.90 (0.86-0.94) | 0.87 (0.84-0.90) | 0.90 (0.87 - 0.93) | 0.72 (0.61 - 0.83) | 0.95 (0.93 - 0.97) | 0.53 (0.42 - 0.63) | 0.3 |
