# Supplementary material for: Predictors and nomogram for amputation risk in pit viper snakebite envenoming at hospital admission
Source: Sci Rep. 2025 Nov 7;15:39082. doi: 10.1038/s41598-025-26903-3 (PMC12594999; doi:10.1038/s41598-025-26903-3)
Supplement: Supplementary file 2 — Supplementary Material 2 [file 41598_2025_26903_MOESM2_ESM.docx]

**Table S5.**

Analytical Performance of Key Biomarkers

| Parameter | Method | Precision (CV%) | Linear Range | Stability | Accuracy Verification |
| --- | --- | --- | --- | --- | --- |
| NLR | Automated CBC | WBC:1.8  Lymph:2.3 | N/A | ≤24h（4℃） | Microscopy correlation (r=0.98) |
| D-dimer | Immunoturbidimetry | 4.8 (0.8 mg/L)  3.2 (8.0 mg/L) | 0.5-35 mg/L | ≤8h （RT） | Spike recovery (95-105%) |
| FIB | Clauss method | 3.2 (1.0 g/L)  2.1 (4.5 g/L) | 0.5-12 g/L | ≤8h （4℃） | Reference method (r=0.99) |
