# Supplementary material for: Predictors and nomogram for amputation risk in pit viper snakebite envenoming at hospital admission
Source: Sci Rep. 2025 Nov 7;15:39082. doi: 10.1038/s41598-025-26903-3 (PMC12594999; doi:10.1038/s41598-025-26903-3)
Supplement: Supplementary file 3 — Supplementary Material 3 [file 41598_2025_26903_MOESM3_ESM.docx]

**Table S4.**

Epidemiological Analysis of Snakebite Envenoming

| Variable | Cases,n (%) | Amputation, n (%) | Statistic | *P* |
| --- | --- | --- | --- | --- |
| Geographic |  |  | χ²=4.91 | 0.027 |
| Rural | 1454 (95.2%) | 205 (14.1%) |  |  |
| Urban | 73 (4.8%) | 5 (6.8%) |  |  |
| Peak Season |  |  | χ²=2.15 | 0.142 |
| May-October | 1,409 (92.3%) | 193 (13.7%) |  |  |
| November-April | 118 (7.7%) | 22 (18.6%) |  |  |
| Species |  |  | χ²=0.42 | 0.936 |
| *Gloydius brevicaudus* | 850 (55.7%) | 121 (14.2%) |  |  |
| *Deinagkistrodon acutus* | 320 (21.0%) | 45 (14.1%) |  |  |
| *Protobothrops mucrosquamatus* | 207 (13.5%) | 29 (14.0%) |  |  |
| *Trimeresurus stejnegeri* | 150 (9.8%) | 20 (13.3%) |  |  |
