# Supplementary material for: Predictors and nomogram for amputation risk in pit viper snakebite envenoming at hospital admission
Source: Sci Rep. 2025 Nov 7;15:39082. doi: 10.1038/s41598-025-26903-3 (PMC12594999; doi:10.1038/s41598-025-26903-3)
Supplement: Supplementary file 4 — Supplementary Material 4 [file 41598_2025_26903_MOESM4_ESM.docx]

**Table S1.**

Assessment of Equilibrium Between Training and Testing sets

| **Variables** | **Total (n = 1527)** | **test (n = 459)** | **train (n = 1068)** | **Statistic** | ***P*** |
| --- | --- | --- | --- | --- | --- |
|  |  |  |  |  |  |
| Amputation, n(%) |  |  |  | χ²=0.00 | 0.997 |
| No | 1314 (86.05) | 395 (86.06) | 919 (86.05) |  |  |
| Yes | 213 (13.95) | 64 (13.94) | 149 (13.95) |  |  |
| Gender |  |  |  | χ²=0.17 | 0.676 |
| Femal | 776 (50.82) | 237 (51.63) | 539 (50.47) |  |  |
| Male | 751 (49.18) | 222 (48.37) | 529 (49.53) |  |  |
| Age(years) | 49.00 (32.00, 63.00) | 50.00 (32.00, 64.00) | 48.00 (33.00, 62.00) | Z=-0.22 | 0.830 |
| Diabetes |  |  |  | χ²=0.01 | 0.916 |
| No | 1303 (85.33) | 391 (85.19) | 912 (85.39) |  |  |
| Yes | 224 (14.67) | 68 (14.81) | 156 (14.61) |  |  |
| Limb vascular disease |  |  |  | χ²=0.00 | 0.958 |
| No | 1233 (80.75) | 371 (80.83) | 862 (80.71) |  |  |
| Yes | 294 (19.25) | 88 (19.17) | 206 (19.29) |  |  |
| Bite location |  |  |  | χ²=0.41 | 0.521 |
| Upper limb | 766 (50.16) | 236 (51.42) | 530 (49.63) |  |  |
| Lower limb | 761 (49.84) | 223 (48.58) | 538 (50.37) |  |  |
| Location of the bitten limb |  |  |  | χ²=0.62 | 0.432 |
| Proximal | 805 (52.72) | 249 (54.25) | 556 (52.06) |  |  |
| Distal | 722 (47.28) | 210 (45.75) | 512 (47.94) |  |  |
| Wound depth |  |  |  | χ²=0.26 | 0.612 |
| Epidermal | 770 (50.43) | 236 (51.42) | 534 (50.00) |  |  |
| Muscle | 757 (49.57) | 223 (48.58) | 534 (50.00) |  |  |
| Activity status at the time of bite |  |  |  | χ²=0.00 | 0.986 |
| Sedentary | 779 (51.02) | 234 (50.98) | 545 (51.03) |  |  |
| Active | 748 (48.98) | 225 (49.02) | 523 (48.97) |  |  |
| Time from injury to admission |  |  |  | χ²=0.07 | 0.786 |
| ≤6h | 946 (61.95) | 282 (61.44) | 664 (62.17) |  |  |
| ＞6h | 581 (38.05) | 177 (38.56) | 404 (37.83) |  |  |
| SSS |  |  |  | χ²=2.72 | 0.256 |
| 0-3 | 643 (42.11) | 201 (43.79) | 442 (41.39) |  |  |
| 4-8 | 606 (39.69) | 168 (36.60) | 438 (41.01) |  |  |
| 8-20 | 278 (18.21) | 90 (19.61) | 188 (17.60) |  |  |
| Percentage of limb swelling on admission(%) | 85.00 (52.00, 119.00) | 85.00 (51.00, 119.50) | 86.00 (54.00, 118.00) | Z=-0.45 | 0.651 |
| Tourniquet misuse |  |  |  | χ²=0.03 | 0.853 |
| No | 1063 (69.61) | 318 (69.28) | 745 (69.76) |  |  |
| Yes | 464 (30.39) | 141 (30.72) | 323 (30.24) |  |  |
| Out-of-hospital wound care |  |  |  | χ²=0.80 | 0.372 |
| Right | 712 (46.63) | 222 (48.37) | 490 (45.88) |  |  |
| Wrong | 815 (53.37) | 237 (51.63) | 578 (54.12) |  |  |
| Antivenom injection time |  |  |  | χ²=2.79 | 0.095 |
| ≤6h | 1109 (72.63) | 320 (69.72) | 789 (73.88) |  |  |
| ＞6h | 418 (27.37) | 139 (30.28) | 279 (26.12) |  |  |
| Antivenom dose (vials) | 3 (2-4) | 3 (2-4) | 3 (2-4) | Z=-0.49 | 0.621 |
| Time from admission to first surgery |  |  |  | χ²=5.01 | 0.082 |
| ＜6h | 901 (59.00) | 289 (62.96) | 612 (57.30) |  |  |
| 6-12h | 476 (31.17) | 125 (27.23) | 351 (32.87) |  |  |
| ＞12h | 150 (9.82) | 45 (9.80) | 105 (9.83) |  |  |
| Surgical approach |  |  |  | χ²=1.24 | 0.266 |
| Conventional incision | 685 (44.86) | 196 (42.70) | 489 (45.79) |  |  |
| VSD | 842 (55.14) | 263 (57.30) | 579 (54.21) |  |  |
| Area of necrotic tissue |  |  |  | χ²=3.06 | 0.216 |
| ＜5cm^2^ | 1091 (71.45) | 325 (70.81) | 766 (71.72) |  |  |
| 5-10cm^2^ | 295 (19.32) | 83 (18.08) | 212 (19.85) |  |  |
| ＞10cm^2^ | 141 (9.23) | 51 (11.11) | 90 (8.43) |  |  |
| Depth of necrotic tissue |  |  |  | χ²=0.34 | 0.559 |
| Superficial | 746 (48.85) | 219 (47.71) | 527 (49.34) |  |  |
| Deep | 781 (51.15) | 240 (52.29) | 541 (50.66) |  |  |
| WBC(109/L) | 13.80 (10.90, 16.20) | 13.90 (10.55, 16.30) | 13.80 (11.10, 16.20) | Z=-0.51 | 0.610 |
| PLT(109/L) | 212.90 (140.10, 299.20) | 214.60 (128.35, 304.35) | 211.25 (143.57, 297.40) | Z=-0.29 | 0.775 |
| NLR | 5.00 (3.10, 7.30) | 5.00 (2.90, 7.30) | 5.00 (3.20, 7.23) | Z=-0.64 | 0.519 |
| CRP(mg/L) | 45.50 (27.65, 64.65) | 45.20 (27.60, 65.55) | 45.55 (27.67, 64.40) | Z=-0.06 | 0.950 |
| ALT(U/L) | 63.80 (36.60, 91.55) | 63.80 (35.65, 93.70) | 63.80 (37.20, 90.17) | Z=-0.04 | 0.972 |
| AST(U/L) | 128.80 (67.65, 191.55) | 131.30 (71.05, 189.30) | 128.50 (65.47, 193.33) | Z=-0.42 | 0.672 |
| BUN (mmol/L) | 6.96 (5.00, 8.94) | 6.79 (5.01, 8.93) | 7.02 (4.99, 8.94) | Z=-0.21 | 0.832 |
| Cr (μmol/L) | 107.00 (78.75, 135.55) | 106.70 (77.45, 137.05) | 107.60 (79.95, 135.00) | Z=-0.40 | 0.690 |
| CK (U/L) | 332.20 (204.70, 461.25) | 319.40 (209.90, 468.75) | 334.35 (201.68, 455.13) | Z=-0.11 | 0.909 |
| PT (s) | 18.40 (15.00, 21.60) | 18.50 (15.20, 21.50) | 18.30 (15.00, 21.70) | Z=-0.67 | 0.501 |
| FIB (g/L) | 1.90 (1.30, 2.70) | 1.90 (1.30, 2.60) | 1.90 (1.30, 2.70) | Z=-1.60 | 0.110 |
| D-dimer (mg/L) | 6.10 (3.30, 9.10) | 6.10 (3.20, 9.30) | 6.10 (3.30, 9.00) | Z=-0.30 | 0.768 |
| Z: Mann-Whitney test, χ²: Chi-square test, M: Median, Q₁: 1st Quartile, Q₃: 3rd Quartile, SSS: Snakebite severity scale, WBC: White blood cell, PLT: Platelet, NLR: Neutrophil-to-lymphocyte ratio, CRP: C-reactive protein, ALT: alanine aminotransferase, AST: Aspartate aminotransferase, BUN: Blood urea nitrogen, Cr: Creatinine, CK: Creatine Kinase, PT: Prothrombin time, FIB: Fibrinogen. | | | | | |
