# Supplementary material for: Predictors and nomogram for amputation risk in pit viper snakebite envenoming at hospital admission
Source: Sci Rep. 2025 Nov 7;15:39082. doi: 10.1038/s41598-025-26903-3 (PMC12594999; doi:10.1038/s41598-025-26903-3)
Supplement: Supplementary file 5 — Supplementary Material 5 [file 41598_2025_26903_MOESM5_ESM.docx]

**Table S2.**

Baseline Characteristics of the Training Set

| Variables | Total (n = 1068) | Non-amputantion (n = 919) | Amputation (n = 149) | Statistic | *P* |
| --- | --- | --- | --- | --- | --- |
|  |  |  |  |  |  |
| Gender |  |  |  | χ²=0.02 | 0.887 |
| Femal | 539 (50.47) | 463 (50.38) | 76 (51.01) |  |  |
| Male | 529 (49.53) | 456 (49.62) | 73 (48.99) |  |  |
| Age(years) | 48.00 (33.00, 62.00) | 48.00 (33.00, 62.00) | 52.00 (32.00, 64.00) | Z=-0.91 | 0.361 |
| Diabetes |  |  |  | χ²=0.19 | 0.659 |
| No | 912 (85.39) | 783 (85.20) | 129 (86.58) |  |  |
| Yes | 156 (14.61) | 136 (14.80) | 20 (13.42) |  |  |
| Limb vascular disease |  |  |  | χ²=0.70 | 0.403 |
| No | 862 (80.71) | 738 (80.30) | 124 (83.22) |  |  |
| Yes | 206 (19.29) | 181 (19.70) | 25 (16.78) |  |  |
| Bite location |  |  |  | χ²=2.49 | 0.114 |
| Upper limb | 530 (49.63) | 465 (50.60) | 65 (43.62) |  |  |
| Lower limb | 538 (50.37) | 454 (49.40) | 84 (56.38) |  |  |
| Location of the bitten limb |  |  |  | χ²=0.06 | 0.800 |
| Proximal | 556 (52.06) | 477 (51.90) | 79 (53.02) |  |  |
| Distal | 512 (47.94) | 442 (48.10) | 70 (46.98) |  |  |
| Wound depth |  |  |  | χ²=0.01 | 0.930 |
| Epidermal | 534 (50.00) | 460 (50.05) | 74 (49.66) |  |  |
| Muscle | 534 (50.00) | 459 (49.95) | 75 (50.34) |  |  |
| Activity status at the time of bite |  |  |  | χ²=2.51 | 0.113 |
| Sedentary | 545 (51.03) | 460 (50.05) | 85 (57.05) |  |  |
| Active | 523 (48.97) | 459 (49.95) | 64 (42.95) |  |  |
| Time from injury to admission |  |  |  | χ²=63.15 | <.001 |
| ≤6h | 664 (62.17) | 615 (66.92) | 49 (32.89) |  |  |
| ＞6h | 404 (37.83) | 304 (33.08) | 100 (67.11) |  |  |
| SSS |  |  |  | χ²=1.32 | 0.517 |
| 0-3 | 442 (41.39) | 374 (40.70) | 68 (45.64) |  |  |
| 4-8 | 438 (41.01) | 382 (41.57) | 56 (37.58) |  |  |
| 8-20 | 188 (17.60) | 163 (17.74) | 25 (16.78) |  |  |
| Percentage of limb swelling on admission(%) | 86.00 (54.00, 118.00) | 86.00 (54.00, 118.00) | 88.00 (54.00, 123.00) | Z=-0.40 | 0.691 |
| Tourniquet misuse |  |  |  | χ²=170.64 | <.001 |
| No | 745 (69.76) | 709 (77.15) | 36 (24.16) |  |  |
| Yes | 323 (30.24) | 210 (22.85) | 113 (75.84) |  |  |
| Out-of-hospital wound care |  |  |  | χ²=0.42 | 0.519 |
| Right | 490 (45.88) | 418 (45.48) | 72 (48.32) |  |  |
| Wrong | 578 (54.12) | 501 (54.52) | 77 (51.68) |  |  |
| Antivenom injection time |  |  |  | χ²=131.65 | <.001 |
| ≤6h | 789 (73.88) | 736 (80.09) | 53 (35.57) |  |  |
| ＞6h | 279 (26.12) | 183 (19.91) | 96 (64.43) |  |  |
| Antivenom dose (vials) | 3 (2-4) | 3 (2-4) | 3 (2-4) | Z=-0.34 | 0.734 |
| Time from admission to first surgery |  |  |  | χ²=0.14 | 0.934 |
| ＜6h | 612 (57.30) | 525 (57.13) | 87 (58.39) |  |  |
| 6-12h | 351 (32.87) | 304 (33.08) | 47 (31.54) |  |  |
| ＞12h | 105 (9.83) | 90 (9.79) | 15 (10.07) |  |  |
| Surgical approach |  |  |  | χ²=1.05 | 0.306 |
| Conventional incision | 489 (45.79) | 415 (45.16) | 74 (49.66) |  |  |
| VSD | 579 (54.21) | 504 (54.84) | 75 (50.34) |  |  |
| Area of necrotic tissue |  |  |  | χ²=0.12 | 0.943 |
| ＜5cm^2^ | 766 (71.72) | 660 (71.82) | 106 (71.14) |  |  |
| 5-10cm^2^ | 212 (19.85) | 181 (19.70) | 31 (20.81) |  |  |
| ＞10cm^2^ | 90 (8.43) | 78 (8.49) | 12 (8.05) |  |  |
| Depth of necrotic tissue |  |  |  | χ²=0.95 | 0.329 |
| Superficial | 527 (49.34) | 459 (49.95) | 68 (45.64) |  |  |
| Deep | 541 (50.66) | 460 (50.05) | 81 (54.36) |  |  |
| WBC(109/L) | 13.80 (11.10, 16.20) | 13.80 (11.35, 15.95) | 14.90 (9.70, 17.60) | Z=-0.67 | 0.503 |
| PLT(109/L) | 211.25 (143.57, 297.40) | 213.60 (147.40, 298.15) | 202.70 (128.30, 285.00) | Z=-1.58 | 0.113 |
| NLR | 5.00 (3.20, 7.23) | 4.90 (3.10, 6.95) | 6.40 (3.80, 8.10) | Z=-4.80 | <.001 |
| CRP(mg/L) | 45.55 (27.67, 64.40) | 45.30 (27.90, 64.15) | 47.00 (26.10, 67.40) | Z=-0.51 | 0.607 |
| ALT(U/L) | 63.80 (37.20, 90.17) | 63.70 (37.10, 91.45) | 64.20 (39.00, 86.10) | Z=-0.23 | 0.815 |
| AST(U/L) | 128.50 (65.47, 193.33) | 128.30 (64.60, 192.25) | 130.40 (90.50, 196.90) | Z=-1.62 | 0.105 |
| BUN (mmol/L) | 7.02 (4.99, 8.94) | 6.96 (5.00, 8.87) | 7.39 (4.84, 9.08) | Z=-0.77 | 0.444 |
| Cr (μmol/L) | 107.60 (79.95, 135.00) | 106.80 (78.55, 135.10) | 110.60 (91.50, 133.00) | Z=-1.26 | 0.209 |
| CK (U/L) | 334.35 (201.68, 455.13) | 333.20 (199.20, 449.95) | 353.00 (206.10, 497.80) | Z=-2.12 | 0.034 |
| PT (s) | 18.30 (15.00, 21.70) | 18.40 (15.00, 21.70) | 17.80 (15.00, 21.40) | Z=-0.29 | 0.768 |
| FIB (g/L) | 1.90 (1.30, 2.70) | 1.90 (1.30, 2.80) | 1.80 (1.40, 2.20) | Z=-2.16 | 0.031 |
| D-dimer (mg/L) | 6.10 (3.30, 9.00) | 5.90 (3.20, 8.90) | 7.20 (4.80, 10.00) | Z=-4.19 | <.001 |
| Z: Mann-Whitney test, χ²: Chi-square test, M: Median, Q₁: 1st Quartile, Q₃: 3rd Quartile, SSS: Snakebite severity scale, WBC: White blood cell, PLT: Platelet, NLR: Neutrophil-to-lymphocyte ratio, CRP: C-reactive protein, ALT: alanine aminotransferase, AST: Aspartate aminotransferase, BUN: Blood urea nitrogen, Cr: Creatinine, CK: Creatine Kinase, PT: Prothrombin time, FIB: Fibrinogen. | | | | | |
